# Electrochemical Determination of Nitrite by Au Nanoparticle/Graphene-Chitosan Modified Electrode

**DOI:** 10.3390/s18071986

**Published:** 2018-06-21

**Authors:** Rijian Mo, Xuehua Wang, Qiong Yuan, Xiemin Yan, Tiantian Su, Yanting Feng, Lulu Lv, Chunxia Zhou, Pengzhi Hong, Shengli Sun, Zhe Wang, Chengyong Li

**Affiliations:** 1 College of Food Science and Technology, Guangdong Ocean University, Zhanjiang 524088, China; rijian_mo@163.com (R.M.); yuanqiong0415@163.com (Q.Y.); xiemin_y@163.com (X.Y.); sutiantiangdou@126.com (T.S.); yanting_feng@163.com (Y.F.); chunxia.zhou@163.com (C.Z.); hongpengzhi@126.com (P.H.); 2 School of Materials Science and Engineering, Wuhan Institute of Technology, Wuhan 430073, China; wang_xuehua@yahoo.com; 3 School of Chemistry and Environment, Guangdong Ocean University, Zhanjiang 524088, China; nhlvluluaio@163.com (L.L.); xinglsun@126.com (S.S.); 4 Shenzhen Institute of Guangdong Ocean University, Shenzhen 518108, China; 5 Coastal Ecology Engineering Technology Research Center of Zhanjiang City, Guangdong Ocean University, Zhanjiang 524088, China; 6 Center for Biomedical Materials and Interfaces, Shenzhen Institutes of Advanced Technology, Chinese Academy of Sciences, Shenzhen 518055, China; zhe.wang@siat.ac.cn

**Keywords:** nitrite, electrochemical sensor, glassy carbon electrode, graphene, chitosan, Au nanoparticles

## Abstract

A highly sensitive nitrite (NO_2_^−^) electrochemical sensor is fabricated using glassy carbon electrode modified with Au nanoparticle and grapheme oxide. Briefly, this electrochemical sensor was prepared by drop-coating graphene oxide-chitosan mixed film on the surface of the electrode and then electrodepositing a layer of Au nanoparticle using cyclic voltammetry. The electrochemical behavior of NO_2_^−^ on the sensor was investigated by cyclic voltammetry and amperometric i-t curve. The results showed that the sensor exhibited better electrocatalytic activity for NO_2_^−^ in 0.1 mol/L phosphate buffer solution (PBS) (pH 5.0). The oxidation peak current was positively correlated with NO_2_^−^ concentration in the ranges of 0.9 µM to 18.9 µM. The detection limit was estimated to be 0.3 µM. In addition, the interference of some common ions (e.g., NO_3_^−^, CO_3_^2−^, SO_4_^2−^, Cl^−^, Ca^2+^ and Mg^2+^) and oxidizable compound including sodium sulfite and ascorbic acid in the detection of nitrite was also studied. The results show that this sensor is more sensitive and selective to NO_2_^−^. Therefore, this electrochemical sensor provided an effective tool for the detection of NO_2_^−^.

## 1. Introduction

Nitrite has been used diffusely in the food industry and other fields [1,2,3]. However, nitrite is detrimental to human health [4,5,6,7]. When its concentration is high in the blood, it can combine with hemoglobin. In this case, the oxygen delivery capacity of hemoglobin will be severe restricted [1,8,9]. Lethal concentrations of NO_2_^−^ intake proposed by the World Health Organization (WHO) are in the range of 8.7 × 10^−6^–2.83 × 10^−5^ mol/L [2]. Therefore, it is critical to create a simple, rapid and sensitive way to detect NO_2_^−^.

Heretofore, there are many methods to detect NO_2_^−^, including chemiluminiscence [10,11], capillary electrophoresis [12], chromatography [13], spectrophotometry [14,15] and electrochemical techniques [7,16]. Among them, the electrochemical techniques, due to their convenient operation, low cost, fast response and high sensitivity, have aroused wide interest. Nevertheless, the conventional electrode has large electrochemical oxidation overpotential during the detection of NO_2_^−^, and the electrode has the possibility of being destroyed by the products produced during the electrochemical process [5,9,17]. In order to resolve these problems, Antonella Curulli et al. prepared a platinum electrode modified with cellulose acetate or poly(1,8-diaminonaphthalene) film, which enables simple and rapid detection of nitrite and nitrate [18]. Since then, the modification of working electrodes by nanomaterials has attracted the attention of researchers [19,20,21]. In particular, Au nanoparticles are widely used for the determination of nitrite due to their biocompatibility, unique structures, excellent conductivity and great electrocatalytic ability [22,23]. For example, Jiang et al. made a biosensor with graphene and gold nanoparticles to detect NO_2_^−^. This sensor has the advantages of high sensitivity and wide concentration range [24]. Li et al. fabricated a sensor to detect NO_2_^−^, which consists of gold nanoparticles and sulfonated graphene. The sensor has the many advantages, such as high sensitivity, wide range of concentration, high selectivity [25]. Rao et al. used CTAB-stabilized Au nanoparticles (AuNPs) and Nafion to fabricate the electrochemical biosensor for detecting NO_2_^−^. This sensor has the advantages of high sensitivity and fast response time [1]. 

In present work, a simple NO_2_^−^ electrochemical sensor was fabricated by drop-coating graphene oxide-chitosan (GO-CS) mixed film on the glassy carbon electrode (GCE) and then electrodepositing a layer of Au nanoparticles (AuNPs) using cyclic voltammetry. Graphene oxide has prominent electronic properties, large specific surface area and excellent stability. Chitosan, a deacetylated derivative of chitin, is rich in amino groups and exhibits good biocompatibility. This sensor has good electrocatalytic activity, large specific surface area and high sensitivity. This electrochemical sensor provided an effective tool for the detection of NO_2_^−^.

## 2. Materials and Methods

### 2.1. Reagents and Materials

Graphite powder, sodium nitrite, Chlorine acid and chitosan (CS, deacetylation, 95%) were purchased from Guangzhou Zuoke Biotechnology Development Co., Ltd. (Guangzhou, China). Ag/AgCl electrode, platinum electrode, glassy carbon electrode (GCE, Φ = 3 mm, CHI104) purchased from Shanghai Chenhua Instrument Co., Ltd. (Shanghai, China). An Ag/AgCl electrode was used as a reference electrode, a platinum (Pt) electrode was used as an auxiliary electrode, and a glassy carbon electrode was used as a working electrode to constitute a three-electrode system. Na_2_HPO_4_, KH_2_PO_4_, and other reagents were purchased from Tianjin Kemiou Chemical Reagent Co., Ltd. (Tianjin, China). The phosphate buffer solution (PBS, 0.1 M, pH 5.0) was obtained by blended Na_2_HPO_4_ and KH_2_PO_4_ solutions and adjusting the pH of the hybrid solution to 5.0. Hydrochloric acid and other chemical reagents were analytical grade, which can be used directly, and we used double distilled water to prepare all solutions.

### 2.2. Preparation of GO

The preparation of GO was according to modified Hummers method [26,27]. Briefly, K_2_S_2_O_8_ (1 g), P_2_O_5_ (1 g) and graphite (2 g) were added into round bottomed flask (250 mL), and sulfuric acid (98%, 10 mL) were slowly added, then heated at 80 °C for 6 h. After that, the mixture was cooled to room temperature and diluted with distilled water carefully. Then, the preoxidation product was obtained by vacuum filtration under reduced pressure to neutral, sonicated in acetone solution for 1 h, and dried in a ventilated kitchen. Afterwards, preoxidized graphene (0.2 g) were added to ice concentrated sulfuric acid (4.6 mL), potassium permanganate (KMnO_4_, 0.6 g) were added slowly with magnetic stirring, stirring at 35 °C for 2 h, and added deionized water (9.2 mL). Then deionized water (28 mL) and 0.5 mL hydrogen peroxide solution (30%) were added. When the color of the solution turned to yellow with stirring, the reaction ended. GO was obtained by centrifugation (3000 rpm, 30 min). The crude GO was cleaned with hydrochloric acid (V:V = 1:10, 50 mL) to remove metal ions, dialyzing it till the dialysate was neutral. The dialyzed to neutral GO water sol was dried by freeze-drying to give dry GO. At last, the dialyzed to neutral GO water sol was dried by freeze-drying to obtain dried GO.

### 2.3. Preparation of GO-CS Solution

The GO solution was obtained by suspending the dried GO in water and sonicated for 12 h. Then the GO solution was centrifuged (3000 rpm, 30 min) to remove the unexfoliated GO.

GO solution (1 mL) was dispersed in 1 mL of 0.2% CS solution (dissolved in acetic acid, pH 4.05) with ultrasonic treatment for 1 h in order to prepare homogeneous GO-CS solution.

### 2.4. The Preparation and Modification of Electrode

**Scheme 1:**
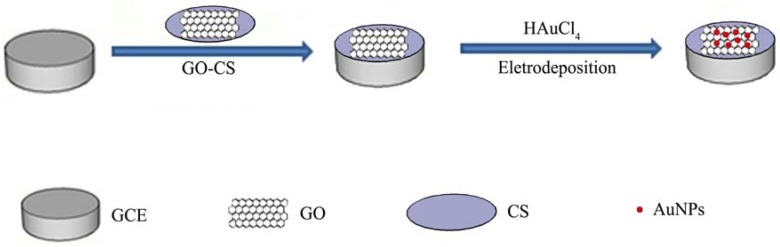
The construction process of graphene oxide (GO)-chitosan (CS)-Au nanoparticles (AuNPs)/glassy carbon electrode (GCE).

First, 0.05 μm alumina slurry was used to polish the surface of glassy carbon electrode (GCE). Next, the electrode was ultrasonicated with ethyl alcohol (95%), KOH (2.0 M), H_2_SO_4_ (1.0 M), ethyl alcohol (95%) and ultrapure water for 3 min in turn. Hereafter, the electrode was cleaned by using double distilled water and dried at natural conditions. The preparation of the modified electrode was as follows: firstly, 5 µL of the GO-CS solution was dropped on the pretreated GCE and dried in air at room temperature. After that, the GO-CS electrode was dipped in the solution which consists of 0.01 M K_2_SO_4_ and 0.04% HAuCl_4_ for 30 min. Finally, the cyclic voltammetry potential was set to −0.6 V–1.6 V. The gold nanoparticles were deposited on the GO-CS electrode surface by cyclic voltammetry. To make a comparison, GO-CS/GCE was prepared by using the similar procedure. The preparation process of the GO-CS-AuNPs/GCE is shown in Scheme 1.

### 2.5. Ion Interference Experiment of Amperometric i-t Curve

To investigated whether some common ions (e.g. NO_3_^−^, CO_3_^2−^, SO_4_^2−^, Cl^−^, Ca^2+^, Mg^2+^ and some oxidizable compounds including sodium sulfite and ascorbic acid) interfere with the modified electrodes for detecting nitrite. The supporting electrolyte and magnetic stirring speeds were 0.1 M PBS (pH 5.0) and 200 r/min, respectively. 10 μL of test solution (1 mM) was added every 50 s. Specifically, NO_2_^−^ solution was added at 50 s, 100 s, 250 s, 300 s, 450 s, 500 s, 650 s, 700 s, 850 s, 900 s, 1050 s, 1100 s, 1250 s and 1300 s. NaCl solution was added at 150s and 200s. Na_2_SO_4_ solution was added at 350 s and 400 s. NaNO_3_ solution was added at 550 s and 600 s. 750 s and 800 s were sequentially added to the Na_2_CO_3_ solution. CaCl_2_ solution was added at 950 s and 1000 s. MgCl_2_ solution was added at 1150 s and 1200 s. Moreover, the oxidizable compound interferes with the sensor also being studied. 10 μL of test solution (1 mM) was added every 50 s. NO_2_^−^ solution was added at 50 s and 100 s. The solution of sodium sulfite was added at 150 s and 200 s. The solution of ascorbic acid was added at 250 s and 300 s.

### 2.6. Instrumentation

Scanning electron microscopy (SEM, Hitachi S-4800) was used to measure the surface morphology of the sample at an accelerating voltage of 15 kV. The thickness of samples was tested via atomic force microscopy (AFM, Veeco-Multimode-V, Plainview, NY, USA). X-ray diffraction (XRD) patterns were reported on a powder X-Ray diffractometer (D8 ADVANCE, Bruker, Germany) with Cu Kα (λ = 0.154 nm) radiation. Transmission electron microscopy (TEM) and high-resolution TEM were measured on an FEI Tecnai G20 at 120 and 200 kV acceleration voltage, respectively.

All electrochemical tests were operated at a CHI 660E electrochemical workstation (CH Instruments, Austin, TX, USA) with a classical three-electrode electrochemical cell. Among them, the working electrode was modified electrode or glassy carbon electrode (GCE), the counter electrode was platinum disk electrode, and the reference electrode was saturated calomel electrode (SCE).

## 3. Results and Discussion

### 3.1. Characterization of GO, GO-CS and GO-CS-AuNPs

**Figure 1:**
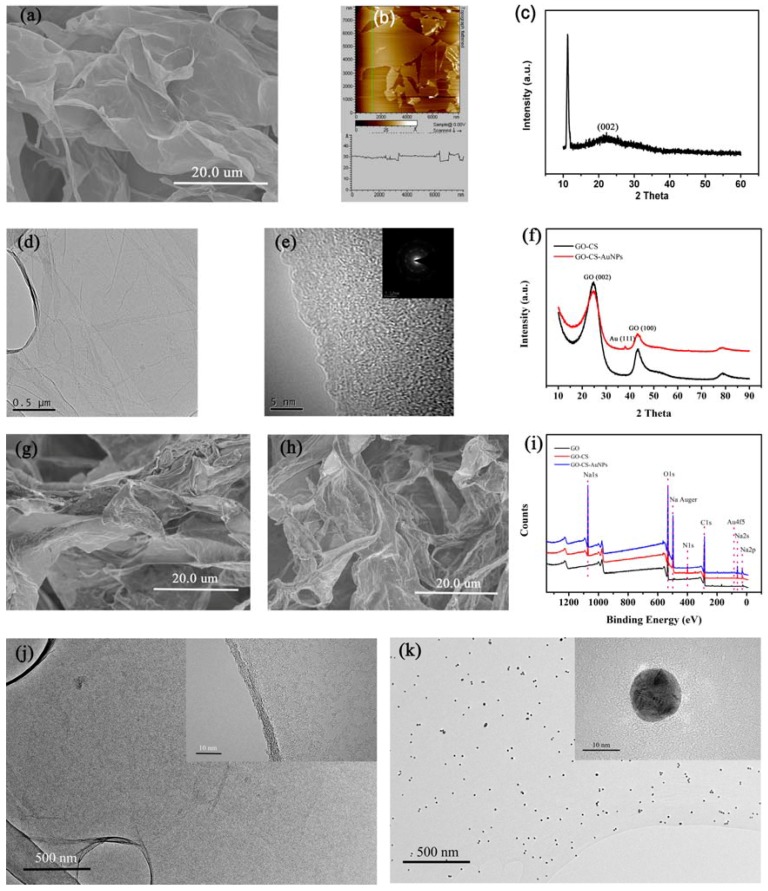
Characterization of GO, GO-CS and GO-CS-AuNPs. (**a**) Scanning electron microscopy (SEM) image of GO; (**b**) Typical atomic force microscopy (AFM) image of GO; (**c**) X-ray diffraction (XRD) pattern of GO; (**d**) Low-resolution transmission electron microscopy (TEM) image of GO; (**e**) High-resolution TEM image and selected area electron diffraction (SAED) pattern (inset) of GO; (**f**) XRD pattern of GO-CS and GO-CS-AuNPs; (**g**) SEM image of GO-CS; (**h**) SEM image of GO-CS-AuNPs; (**i**) X-ray photoelectron spectroscopy (XPS) of GO, GO-CS and GO-CS-AuNPs; (**j**) Low-resolution TEM image and High-resolution TEM image (inset) of GO-CS; (**k**) Low-resolution TEM image and High-resolution TEM image (inset) of GO-CS-AuNPs.

The characteristics of GO, GO-CS and GO-CS-AuNPs are shown in Figure 1. The scanning electron microscopy (SEM) of GO is shown in Figure 1a, unveiling the representative wrinkled and puckery graphene lamellate structure. The morphology and layers of GO can be characterized by atomic force microscopy (AFM). The GO’s AFM image is shown in Figure 1b. The average thickness of the GO sheet stripped by ultrasound is 0.6~1 nm. This is mainly because of the conjugation of many –COOH, –OH and epoxy groups on two flanks of the graphene nanoscale in the process of oxidation. Figure 1c shows the X-ray diffraction (XRD) pattern of GO. GO’s diffraction peak indexed to (002) shifts negatively to ~11.2°, corresponding to a layered structure with a basal spacing of 0.79 nm. The morphology and structure of the prepared GO were observed by transmission electron microscopy (TEM). Figure 1d shows the low-magnification TEM image of GO. GO nanosheets reveal uniform layered forms such as wrinkled silk surface waves upon its compression and accumulation randomly. High-resolution transmission electron micrograph (HRTEM) and selected area electron diffraction (SAED) of GO are shown in Figure 1e, revealing the GO has great crystallographic structure. Moreover, sharp edges composed of non-defective graphite layers with (002) crystal planes are evident (Figure 1c). It corresponds to a d-spacing of about 0.35 nm, which is near to that of single crystal graphite (0.34 nm). As shown in SAED (inset in Figure 1e), the clearly diffraction spots and loops are completely pointing to the typical hexagonal lattice carbon of GO, which further demonstrates the crystalline nature of GO. Figure 1f shows the X-ray diffraction (XRD) pattern of GO-CS and GO-CS-AuNPs. The GO-CS complex (blank line) shows the (002) plane diffraction peak and the (100) plane of GO at 2θ = 24.9° and 2θ = 43.1°, respectively. However, its broad peak shape indicates that the presence of chitosan has a certain degree of influence on the crystallization of GO. The GO-CS-AuNPs complex (red line) shows the characteristic absorption peak of gold nanoparticles at 38.1°, corresponding to the (111) crystal plane of the cubic system of gold nanoparticles. In addition, the intensity of diffraction peaks of the graphite (002) and (100) planes in the XRD curves of GO-CS-AuNPs is significantly smaller than that of GO-CS. The reason is that the gold nanoparticles loaded on the surface of GO disrupt the ordered structure of GO. The GO-CS composite was characterized using SEM as shown in Figure 1g. The GO-CS composite exhibits a folded, rough and continuous surface, which indicates that GO and CS constitute highly compatible composites. The SEM was used to characterize the GO-CS-AuNPs composites, as shown in Figure 1h. The morphology of GO-CS-AuNPs composites is similar to that of GO-CS. X-ray photoelectron spectroscopy (XPS) proved that CS and AuNPs can form a highly compatible composite with GO, as shown in Figure 1i. The GO powder (black line) has a pronounced O1s and C1s peak, but the N1s peak is not obvious. A clear peak of N 1s was observed on the GO-CS composite (red line). The peak of the Na element is more obvious because of the complex adjustment of the pH using NaOH. The GO-CS-AuNPs composites (blue line) do not have an obvious peak of Au elements due to the small amount of Au nanoparticles. The morphology of GO-CS composite was investigated using TEM. Low-resolution TEM image and High-resolution TEM image (inset) of GO-CS are shown in Figure 1j, the flat morphology of GO-CS complex can be observed. Figure 1k shows the size and morphology of GO-CS-AuNPs. The topological structure of GO nanosheets shows flat with a few layered sheets and transparent. In addition, the spherical of Au nanoparticles can be observed on the composite, which indicates that GO-CS-AuNPs has been successfully synthesized. As shown in Figure 1k, Au nanoparticles have not uniform in size. The nanoparticle average diameters were calculated at about 14 nm. 

### 3.2. Electrochemical Behavior of NO_2_^−^ on the Electrode

**Figure 2:**
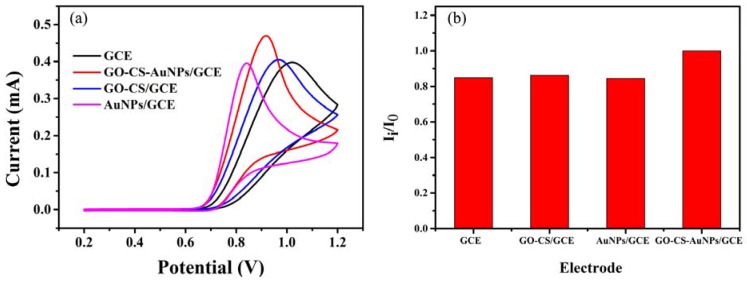
The electrochemical behaviors of GCE, GO-CS/GCE, AuNPs/GCE and GO-CS-AuNPs/GCE in the presence of 10 mM NO_2_^−^. (**a**) Cyclic voltammograms at GCE, GO-CS/GCE and GO-CS-AuNPs/GCE in the presence of 10 mM NO_2_^−^ in the 0.1 M pH 5.0 phosphate buffer solution (PBS) (scan rate: 100 mV/s). (**b**) The ratio of the peak current (I_i_) of the other electrodes to the peak current (I_0_) of GO-CS-AuNPs/GCE in (**a**).

The electrochemical behaviors of 10 mM NO_2_^−^ at the electrodes of GCE, GO-CS/GCE, AuNPs/GCE and GO-CS-AuNPs/GCE are shown in Figure 2. Figure 2a shows that the anodic peak current of NO_2_^−^ is 470 µA at the GO-CS-AuNPs/GCE, which is strongest compared to the others. Figure 2b shows the ratio of the peak current (I_i_) of the other electrodes to the peak current (I_0_) of GO-CS-AuNPs/GCE in Figure 2a. The peak current of GCE, GO-CS/GCE and AuNPs/GCE are about 0.85, 0.86 times and 0.84 times of GO-CS-AuNPs/GCE, respectively.

From the abovementioned phenomena, it can be concluded that GO-CS-AuNPs/GCE exhibits good electrochemical catalytic activity for the detection of NO_2_^−^, which was accounted for by the following factors. Firstly, the redox activity of NO_2_^−^ on modified electrode generally improved by small size effect and surface effect of graphene. Secondly, since its protonation of –NH_2_ in the polymer in acidic solutions, CS presents as a positively charged polymer. On the electrode surface, the protonated –NH_2_ may attract NO_2_^−^, which leads to the enhancement of response currents [28]. Thirdly, the dispersed Au nanoparticles on the surface of GO-CS composites provides a way for the electron transfer and catalytic process, because it enhances the contact area of the catalytic reaction [29]. Furthermore, Au nanoparticles on the surface of GO-CS composites can be combined with NO_2_^−^ to form Au-N bonds, which enhance the response current [20]. Therefore, GO-CS-AuNPs/GCE exhibited a better response for detection of NO_2_^−^.

### 3.3. The Effect of pH on NO_2_^−^ Detection

**Figure 3:**
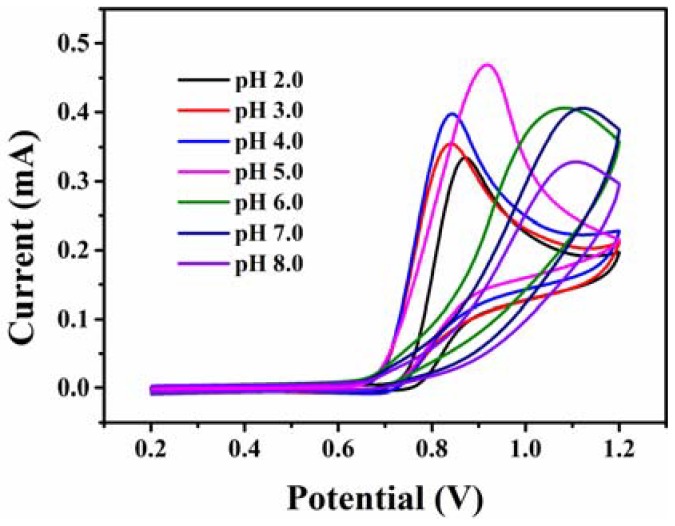
Effects of 0.1 M PBS with different pH (2.0~8.0) on the detection of NO_2_^−^ by modified electrode.

The effect of pH on the NO_2_^−^ detection by GO-CS-AuNPs/GCE was studied using cyclic voltammetry, as depicted in Figure 3. The potential of the oxidation peak gradually shifted with pH from 2.0 to 8.0. The peak current increases with the increase of pH when the pH is less than 5.0. However, when the pH is higher than 5.0, the peak current decreases as the pH increases. The current response is the strongest at pH 5.0. Therefore, pH 5.0 of PBS solution (0.1 M) was selected as a support electrolyte solution for the quantitative detection of nitrite.

### 3.4. The Effect of Scan Rate on NO_2_^−^ Detection

**Figure 4:**
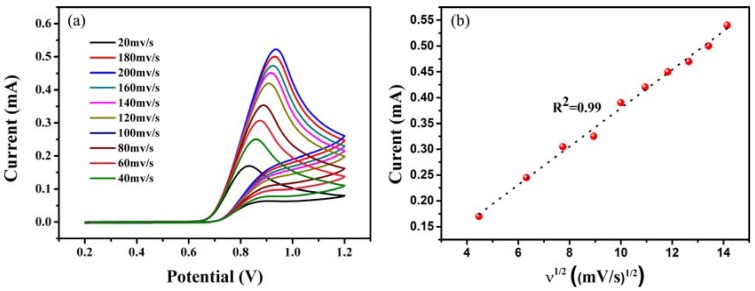
Effect of scan rate on detection of nitrite by modified electrode; (**a**) Cyclic voltammograms of 10 mM NO_2_^−^ at GO-CS-AuNPs/GCE in 0.1 M pH 5.0 PBS at the scan rates of 20, 40, 60, 80, 100, 120, 140, 160, 180 and 200 mV/s, respectively; (**b**) The current response is positively correlated with the square roof of the scan rate.

The effect of scan rate on the electrochemical behavior of NO_2_^−^ in 0.1 M PBS (pH 5.0) at the GO-CS-AuNPs/GCE is shown in Figure 4. Figure 4a shows the oxide peak currents increase with the scan rate increasing from 20 mV/s to 200 mV/s. Figure 4b shows the linear correlation between the NO_2_^−^ peak current and the square root of the scan rate, the correlation coefficient reached 0.99. The outcomes suggest that the dynamics of the total course is controlled by diffusion.

### 3.5. Interference Study

**Figure 5:**
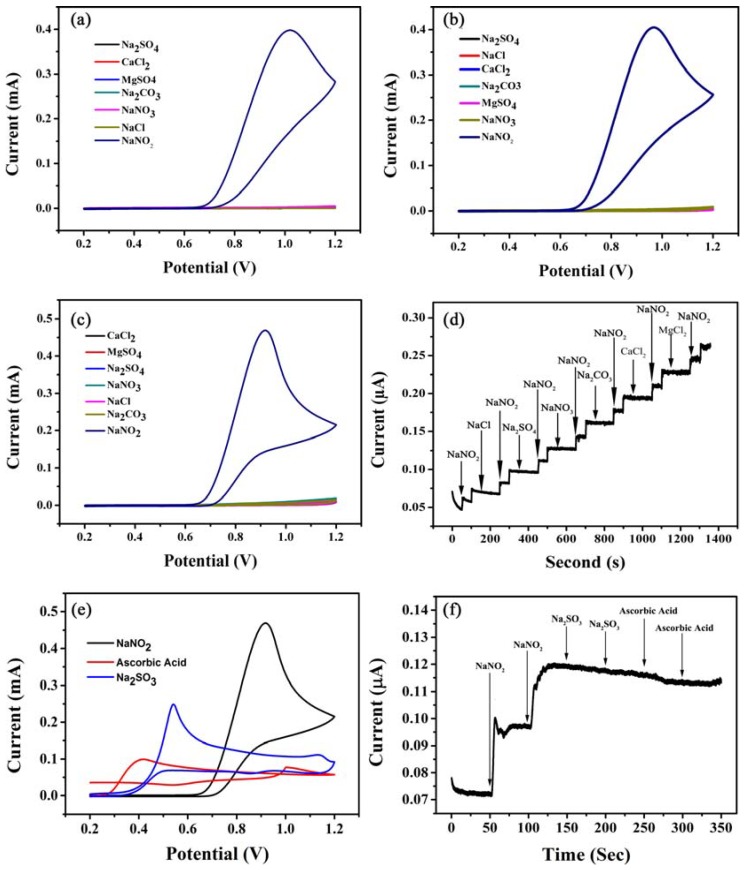
Effect of common ions on different modified electrodes; (**a**) Cyclic voltammograms of 10 mM NO_2_^−^, NO_3_^−^, CO_3_^2−^, SO_4_^2−^, Cl^−^, Ca^2+^, Mg^2+^ at GCE in 0.1 M pH 5.0 with 100 mV/s; (**b**) Cyclic voltammograms of 10 mM NO_2_^−^, NO_3_^−^, CO_3_^2−^, SO_4_^2−^, Cl^−^, Ca^2+^, Mg^2+^ at GO-CS/GCE in 0.1 M pH 5.0 with 100 mV/s; (**c**) Cyclic voltammograms of 10 mM NO_2_^−^, NO_3_^−^, CO_3_^2−^, SO_4_^2−^, Cl^−^, Ca^2+^, Mg^2+^ at GO-CS-AuNPs/GCE in 0.1 M pH 5.0 with 100 mV/s; (**d**) Amperometric response of biosensor to successive additions of different concentration in 0.1 M pH 5.0 PBS with working potential of 0.8 V. (**e**) Cyclic voltammograms of 10 mM sodium nitrite, sodium sulfite and ascorbic acid at GO-CS-AuNPs/GCE in 0.1 M pH 5.0 with 100 mV/s; (**f**) Amperometric i-t curve of sodium nitrite, sodium sulfite and ascorbic acid detected by GO-CS-AuNPs/GCE biosensor in 0.1 M pH 5.0 PBS at 0.8 V of working potential.

The potential interference of some common ions on the detection of NO_2_^−^ was studied using cyclic voltammetry. Figure 5a shows that no interference was observed for 10 mM NO_2_^−^ in the presence of 10 mM of NO_3_^−^, CO_3_^2−^, SO_4_^2−^, Cl^−^, Ca^2+^ and Mg^2+^ at the bare GCE. Figure 5b shows that no interference was observed for 10 mM NO_2_^−^ in the presence of 10 mM of NO_3_^−^, CO_3_^2−^, SO_4_^2−^, Cl^−^, Ca^2+^ and Mg^2+^ at the same concentrations of NO_2_^−^ at the GO-CS/GCE. Figure 5c shows that no interference was observed for 10 mM NO_2_^−^ in the presence of 10mM of NO_3_^−^, CO_3_^2−^, SO_4_^2−^, Cl^−^, Ca^2+^ and Mg^2+^ at the same concentrations of NO_2_^−^ at the GO-CS-AuNPs/GCE. In addition, the results show that GO-CS-AuNPs/GCE is more sensitive to NO_2_^−^ and its anti-interference ability is better.

In order to further demonstrate the anti-interference ability and selectivity of GO-CS-AuNPs/GCE. The same concentration of NO_2_^−^ and interfering ions (e.g., NO_3_^−^, CO_3_^2−^, SO_4_^2−^, Cl^−^, Ca^2+^ and Mg^2+^) were detected using an amperometric i-t curve. GO-CS-AuNPs/GCE was used as the working electrode, and the potential is 0.8 V. The current increases when NO_2_^−^ is added, but the current does not change significantly when NO_3_^−^, CO_3_^2−^, SO_4_^2−^, Cl^−^, Ca^2+^ and Mg^2+^are added as shown in Figure 5d. Therefore, NO_3_^−^, CO_3_^2−^, SO_4_^2−^, Cl^−^, Ca^2+^ and Mg^2+^ did not affect the detection of NO_2_^−^ by GO-CS-AuNPs/GCE. In summary, the sensor has good selectivity and anti-interference ability.

To demonstrate the selectivity of the sensor in the presence of oxidizable compound, the GO-CS-AuNPs/GCE biosensor for the detection of sodium nitrite, sodium sulfite and ascorbic acid are shown in Figure 5e,f. Figure 5e shows the cyclic voltammograms of sodium nitrite (10 mM), sodium sulfite (10 mM) and ascorbic acid (10 mM) were detected by the GO-CS-AuNPs/GCE biosensor. The oxidation reaction of sodium nitrite, sodium sulfite and ascorbic acid can be catalyzed by the biosensor, but the oxidation potential is different. To further determine the selectivity of the sensor for sodium nitrite, the same concentrations of the sodium nitrite, sodium sulfite and ascorbic acid were detected by the amperometric i-t curve at the potential of 0.8 V as shown in Figure 5f. After sodium nitrite was added, the current response was significantly increased, whereas the current response did not change significantly when sodium sulfite and ascorbic acid were added. Therefore, the sensor has good selectivity for nitrite.

### 3.6. The Detection of NO_2_^−^

**Figure 6:**
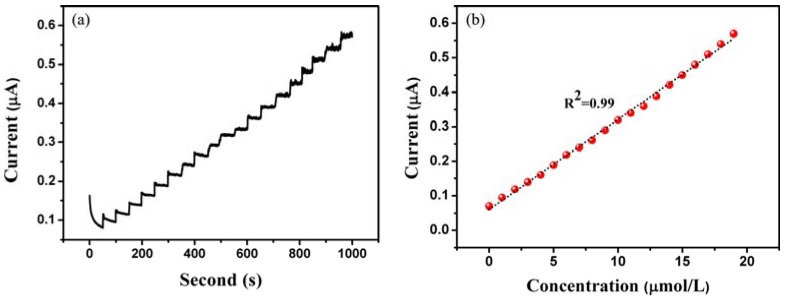
Detection of NO_2_^−^; (**a**) Amperometric response of biosensor to successive additions of concentration NO_2_^−^ in 0.1 M PBS (pH 5.0); (**b**) The linear relation of amperometric response of GO-CS-AuNPs/GCE.

Figure 6a exhibits the electrocatalytic oxidation behavior of NO_2_^−^ at the potential of 0.8 V, and 10 µL of 1 µM NO_2_^−^ was added each 50 s during the measurement. Evidently, the current of GO-CS-AuNPs/GCE increased with increasing NO_2_^−^ concentration in PBS solution (0.1 M, pH 5.0). The calibration curve of the current vs. concentration was obtained using data from these measurements. As shown in Figure 6b, the current is linearly with the concentration of NO_2_^−^ in the ranges of 0.9 µM to 18.9 µM. The linear regression equation was I = 0.2612C + 0.6025 (R^2^ = 0.99). The limit of detection defined as LOD = 3SD/k, where LOD, SD and k are the limit of detection, standard deviation of the blank and the slope of the calibration graph, respectively, was found to be about 0.3 µM. 

The linear range and detection limit of other biosensors were compared to those of the sensor we proposed, GO-CS-AuNPs/GCE. The results are displayed in Table 1. Although the analytical performance of GO-CS-AuNPs/GCE is slightly weaker than these reported biosensors [2,22,24,30,31,32], the preparation process of GO-CS-AuNPs/GCE is relatively simple and the selectivity is high. Moreover, the detection limit of our proposed GO-CS-AuNPs/GCE is lower than other reported biosensors [33,34,35,36,37,38].

## 4. Conclusions

In conclusion, we fabricated a simple and sensitive NO_2_^−^ electrochemical sensor constructed with GO, CS and Au nanoparticle composite modified electrode. Under the optimal conditions, the GO-CS-AuNPs/GCE exhibited a good electrochemical response to NO_2_^−^, owing to the surface effect of GO, the protonated -NH_2_ which, on the surface of CS, attracts negatively charged ions (NO_2_^−^), and the gold nanoparticles that could bond to NO_2_^−^ to form Au-N key. The sensor showed an excellent electroanalytical feature towards NO_2_^−^. The oxidation peak current was positively correlated with NO_2_^−^ concentration in the ranges of 0.9 µM to 18.9 µM and the linear correlation coefficient reached 0.99. The limit of detection is calculated as about 0.3 μM. This method provides a valid method for detecting NO_2_^−^ and is expected to be used for the detection of NO_2_^−^ in foods.

## Figures and Tables

**Table 1:** Comparison of several biosensors with the proposed biosensor for detecting NO_2_^−^.

Biosensors	Linear Range (μM)	Detection Limit (µM)	References
GO-CS-AuNPs/GCE	0.9–18.9	0.3	This study
Pd/RGO/GCE	1–1000	0.23	[22]
Ag/HNTs/MoS_2_-CPE	2–425	0.7	[33]
Hb/Au/GACS/GCE	0.05–1000	0.01	[24]
NGE/PdNC/GCE	0.5–1510	0.11	[2]
CR-GO/GCE	8.9–167	1	[34]
Co_3_O_4_/RGO/GCE	1–380	0.14	[30]
AgNP/GC	10–1000	1.2	[37]
GNPs/MWCPE	0.05–250.0	0.01	[31]
Cu-NDs/RGO/GCE	1.25–13,000	0.4	[35]
Ag-PAMAM nanocomposite/GCE	4–1400	0.4	[36]
Au-Fe(III) nanoparticle/GCE	0.3–150	0.2	[32]
poly(o-anisidine) ionic liquid carbon paste electrode	2–50	1.05	[38]
